# Effect of femoral neck modularity upon the prosthetic range of motion in total hip arthroplasty

**DOI:** 10.1007/s11517-014-1171-9

**Published:** 2014-06-27

**Authors:** Glen A. Turley, Damian R. Griffin, Mark A. Williams

**Affiliations:** 1 WMG, The University of Warwick, Coventry, CV4 7AL UK; 2 Warwick Medical School, The University of Warwick, Coventry, CV2 2DX UK

**Keywords:** Total hip arthroplasty, Femoral neck modularity, Impingement, Range of motion

## Abstract

In total hip arthroplasty, aseptic loosening and dislocation are associated with not being able to achieve the correct prosthetic component orientation. Femoral neck modularity has been proposed as a solution to this problem by allowing the surgeon to alter either the neck-shaft or version angle of the prosthetic femoral component intra-operatively. A single replicate full factorial design was used to evaluate how effective a modular femoral neck cementless stem was in restoring a healthy prosthetic range of motion in comparison with a leading fixed-neck cementless stem with the standard modular parameters. It was found that, if altered to a large enough degree, femoral neck modularity can increase the amount of prosthetic motion as well as alter its position to where it is required physiologically. However, there is a functional limit to the amount that can be corrected and there is a risk with regard to the surgeon having to select the optimum modular neck before any benefit is realised.

**Electronic supplementary material:**

The online version of this article (doi:10.1007/s11517-014-1171-9) contains supplementary material, which is available to authorized users.

## Introduction

During total hip arthroplasty (THA), being able to control the orientation of the prosthetic components is of critical importance in normalising the biomechanics of the hip [36, 54]. This requires having both a stable joint as well as achieving the ideal range of motion for a patient to fulfil their daily activities [8]. Failure to achieve these outcomes is linked to two of the most prominent reasons for revision surgery, aseptic loosening secondary to wear and dislocation which account for 45 and 15 % of revision cases, respectively [25]. To achieve joint stability, the surgeon is required to make appropriate adjustments in the orientation of the prosthetic components to achieve the required femoral head coverage [17]. However, during daily activities, an overly contained cup increases the risk of impingement, whereby the neck of the femoral component contacts with the rim of the acetabular cup creating wear particles and also micro-motion of the acetabular cup leading to eventual implant loosening [13, 27, 30, 49]. Further motion beyond the impingement point causes subluxation of the femoral head until the joint dislocates [11, 17]. However, orienting the prosthetic components to maximise range of motion by increasing both acetabular cup inclination and the combined version of the acetabular cup and femoral stem would mean only partial containment of the hip joint [31, 54]. This risks both aseptic loosening and joint dislocation whereby the femoral head ‘slips out’ of the acetabular cup [11, 47].

The clinical community have presented recommendations with regard to prosthetic component orientation which ideally balances the trade-off between stability and impingement [15, 19, 32, 47, 49, 55]. Considering the orientation of the acetabular cup, Yoon et al. [53] compared these recommendations and found that it was advised to achieve between 32–50° inclination and 8–25° anteversion when expressed using the radiographic convention [23]. Regarding the version angle of the femoral component, due to the acknowledged interdependence with anteversion of the acetabular cup, combined version values have been posed which range between 25° and 60° [7, 10, 28, 31]. As well as these recommendations, prosthetic design factors such as the head-neck ratio, cup-opening plane, neck-shaft angle and the geometry of the femoral neck also influence the post-operative range of motion [11, 49]. These design factors determine the overall range of motion which a THA can achieve. The orientation of the prosthetic components then interacts to position this range of motion area to where it is required physiologically [49]. Therefore, achieving the correct prosthetic component orientation to achieve both ideal range of motion and secure containment within the constrained prosthetic impingement limits is vital to operative success.

A recent addition to THA prosthetic component design has been the addition of a modular femoral neck which provides the surgeon with the ability to independently adjust femoral neck offset, femoral neck-shaft angle as well as the version angle of the femoral neck [30, 36, 48]. The latter is regarded to be particularly important, as controlling the version angle of the femoral neck can be limited by the chosen fixation method. In Australia, the UK and the USA cementless THA is the method of fixation used in the majority of cases [4, 25]. Consequently, the surgeon has limited flexibility to control the degree of anteversion, in comparison with cemented THA. This is due to the orientation of the femoral stem being influenced by the variable geometry of femoral medullary canal [17]. Therefore, having independent adjustment of the femoral neck may prove advantageous in comparison with fixed femoral neck devices [8, 38].

A number of clinical and experimental studies have demonstrated the effectiveness of femoral neck modularity [8, 20, 30, 31, 36–39]. However, the Australian National Joint Registry found that some modular neck stems had a 5-year cumulative revision rate of 11 % [24]. Further, there have been concerns with regard to the integrity of the modular femoral neck [34, 36]. These concerns relate to the taper fitting of the femoral neck to the femoral stem, which potentially cause excessive fretting and crevice corrosion to the modular neck [12]. This has been linked to recent case reports of modular femoral necks fracturing, which have been hypothesised to be caused by the increased moment arm of the long anteverted modular femoral necks, combined with the functional demands of a heavier patient [34, 50, 51]. This increased stress, along with corrosion and fretting could lead to degradation and failure at the stem-neck junction [12, 50, 51]. The risk of fretting is not particular to the femoral stem-neck junction, and it is also a risk at other taper sites such as at the head-neck junction, particularly with larger diameter heads [44]. However, further experimental work needs to be done to evaluate whether femoral neck modularity offers any benefit over fixed femoral neck devices which could be exploited if its limitations were resolved.

Both fixed-neck and modular neck implants have a range of other modular options, such as choice of femoral head diameter, which have been well reported in being able to maximise the range of motion until impingement [3, 26, 48, 54]. Consequently, femoral neck modularity needs to be assessed in comparison with other THA features with regard to whether they offer any additional benefit in improving range of motion until impingement. In this study, range of motion simulation was used to evaluate whether a modular neck cementless THA system can provide additional benefit with regard to reducing instances of impingement in comparison with a leading fixed femoral neck cementless THA.

## Methods

To evaluate the effectiveness of femoral neck modularity, a full factorial experiment was designed [22]. Two implant types were selected for comparison, the Corail (*Depuy, Warsaw, IN, USA*) straight tapered cementless stem for the non-modular control and the Profemur (*Wright Medical Technology, Arlington, TN, USA*) cementless stem as the modular neck intervention. This selection provided a comparison of femoral neck modularity against a non-modular control which is the most widely implanted cementless stem in the UK, used in 33,724 THA procedures in 2011 [25].

**Fig. 1:**
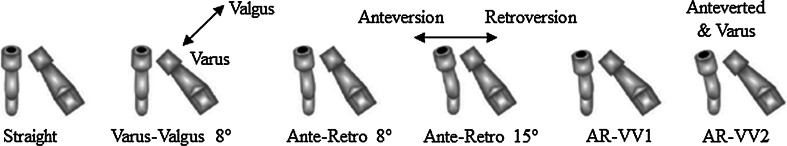
Modular femoral neck options: straight, varus–valgus 8° and 15° (not shown), ante-retroverted 8° and 15°, combination of 4.5° ante–retroversion with 6° of varus–valgus the AR-VV1 and AR-VV2 options Traina et al. [37]

To measure the effect that femoral neck modularity had upon the post-operative impingement free range of motion in comparison with other THA implant parameters, high (+) and low (−) values for each of the implant-specific independent variables were defined, Table 1. Referring to Table 1, for the non-modular neck control group, the acetabular liner rotation centre depth influences the opening angle of the acetabular cup, while the femoral stem length provided an objective scale to indicate the overall size of the implant including the thickness of the femoral neck. Hence, both these factors influenced the range of motion until impingement. Referring to Table 1, for the modular neck intervention group, the femoral neck length affects the impingement point on the tapered femoral neck and therefore range of motion until impingement. Figure 1 provides a schematic of the femoral neck options within the modular neck intervention group. Together with a straight neck option, there were in total nine modular neck options—8º or 15º pure anteverted, retroverted, varus and valgus options as well AR-VV options which combine 4.5º of anteversion or retroversion with 6º of varus or valgus.

**Table 1:** High (+) and low (−) values of the independent variables for the non-modular control group and modular neck intervention group

Femoral head diameter (mm)	Acetabular rotation centre depth (mm)	Femoral head offset (mm)	Femoral neck offset	Femoral neck-shaft angle (°)	Femoral stem length (mm)
*Non*-*modular control group*					
(+) 36	0	+3.5	High offset	125	110
(−) 28	+2	−3.5	Std offset	135	170

Femoral head diameter (mm)	Acetabular rotation centre depth (mm)	Femoral head offset (mm)	Femoral neck offset (mm)	Neck anteversion/retroversion	Modular neck varus/valgus
*Modular neck intervention group*					
(+) 36	0	+3.5	Long (38.5)	15° Ante	15° Varus
(−) 28	+1	−3.5	Short (28)	15° Retro	15° Valgus

As well as implant-specific parameters, the orientation in which the prosthetic components are implanted can also affect the post-operative impingement free range of motion [32, 55]. These orientation parameters were defined, according to the definition provided by Murray [23], as operative acetabular cup inclination, operative acetabular anteversion and anatomical femoral stem version. To obtain an estimate for these parameters, measurement using a prototype surgical tracking system was used (*Brainlab, Feldkirchen, Germany*) of non-navigated THA procedures. These measurements were taken as part of a separate but related study at the University Hospitals Coventry and Warwickshire NHS Trust. A total of 49 (20 males, 29 females) non-modular control hips were measured having a mean age of 65.1 (39–83 years). In the modular neck intervention group, there were 48 (19 males, 29 females) hips having a mean age of 63.6 (23–80 years). The high (+) and low (−) values for each orientation parameter were defined as the pooled ± 2 standard deviation values from the combined 97 intra-operative measurements of both the non-modular and modular neck implant groups—acetabular inclination (mean 37.3º, CI 1.4º, SD 7.1º), acetabular anteversion (mean 22.9º, CI 3.0º, SD 14.5º) and femoral stem version (mean 7.2º, CI 2.8º, SD 13.6º). There were no significant differences (*p* ≤ 0.05) between the component placements of each of the two implant groups, Table 3 [2, 45].

To calculate the prosthetic range of motion, the ±2 standard deviation measured prosthetic orientation values were used to orientate theoretically perfect CAD geometric representations of the THA implants within the 3D environment of the Brainlab Hip Essential 5.1.2 software (*Brainlab, Feldkirchen, Germany*). The acetabular component was orientated relative to the pelvic coordinate frame defined through the palpation of the anterior pelvic plane (APP) landmarks. The femoral component was positioned according to the femoral coordinate frame which was constructed using the ankle epicondyle piriformis (AEP) plane used to define the neutral rotation of the femur [18, 41, 52]. Once the prosthetic components were orientated with respect to their individual body-segment coordinate frames, the femoral coordinate frame was located relative to the pelvic coordinate frame so that the knee centre had a position vector of$P = \left( {0, - 1,0} \right)$. This knee centre position defined the anatomical neutral posture when a person stood upright so that the knee centre lies directly below the hip centre and represented the start point for the range of motion simulation [16, 29].

**Fig. 2:**
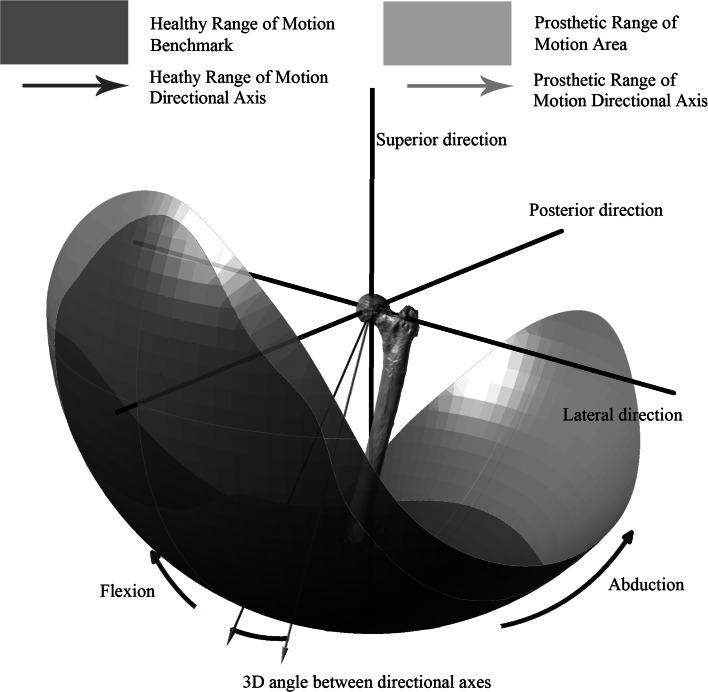
Comparison of the prosthetic motion area (*purple*) against a healthy range of motion benchmark (*gold*). Position of prosthetic motion area evaluated at the three-dimensional angle between its directional axis (*purple*) and the directional axis of healthy range of motion benchmark (*red*) (colour figure online)

The prosthetic range of motion was simulated by virtually rotating the femoral implant about axes in the transverse plane until it collided with the acetabular liner. These rotation axes were constructed according to Eq. 1 [40] where the angle $\alpha$ was stepped in 10° increments in the transverse plane, producing 36 separate axes for the femoral component to be rotated about. These rotation axes were used based on previous findings that many daily activities which pose risk of dislocation such as sitting on low chair, stooping down to pick an object up from the floor or kneeling to tie one’s shoelaces had their axes of rotation within 15° of the transverse plane [40]. The femoral component was rotated about each of the separate rotation axes until collision, or impingement, occurred between the acetabular liner and the neck of the femoral component. Each collision point was then plotted to construct a graphical representation of the prosthetic range of motion, which is shown in purple in Fig. 2, and defined as the ‘prosthetic motion area’. This prosthetic motion area was then compared with a healthy range of motion benchmark, shown in gold in Fig. 2, and defined as the ‘healthy benchmark’. This healthy benchmark was constructed using the same rotation axes used in the prosthetic range of motion simulation, Eq. 1, based on measurements of healthy individuals performing those daily activities which pose risk of dislocation [40]. Two outcome measures were used based on the comparison of the prosthetic motion area with the healthy benchmark (1) the size of the prosthetic motion area *and* (2) the position of the prosthetic motion area. The size of the prosthetic motion area was calculated as a percentage of the surface area of the healthy benchmark. Hence, a prosthetic motion area having an area greater than 100 % would be large enough to provide an impingement free range of motion, providing that it was positioned in an area where it is required physiologically.

The position of the prosthetic motion area was defined by comparing its position relative to the healthy benchmark. This outcome measure was used to evaluate the extent to which each of the THA implant parameters was able to adjust the position of prosthetic motion area. Therefore, using the two outcome measures, a prosthetic motion area that was larger than the healthy benchmark and positioned in such a way that it encompasses the same healthy benchmark would enable a patient to fulfil their daily activities. To define the position of the prosthetic motion area, a directional axis was defined for both the healthy benchmark and the prosthetic motion area [43]. This axis represented the normal vector to a best-fit plane constructed from points taken at the edge of the respective range of motion areas [6, 9]. For the prosthetic motion area, these points represented the impingement point for each of the 36 rotation axes. The $x,y,z$ spatial coordinates of the individual impingement points from the range of motion simulation was used to calculate the centre of mass $\bar{x},\bar{y},\bar{z}$ of the prosthetic range of motion area (Eq. 2). The distance of each of the impingement points away from the calculated centre of mass was then determined (Eq. 3) and arranged in $3 \times n$ matrix, $A$. The dot product $A \cdot A^{T}$ shown in Eq. 4 was solved to find the directional axis of the prosthetic motion area by determining the eigenvector $(\upsilon )$ which maximised the distance to the boundary edge points [6, 9]. The position of the prosthetic motion area was calculated as the three-dimensional angle between its directional axis and the directional axis of the healthy benchmark, Fig. 2. Further illustration of how these outcome measures were developed is provided in the supplementary file which accompanies this study.

1 $$T = \left( {\sin \alpha ,0,\cos \alpha } \right)$$

2 $$\left( {\bar{x},\bar{y},\bar{z}} \right) = \frac{{\left( {\sum\nolimits_{n = 1}^{n} {(x)} ,\sum\nolimits_{n = 1}^{n} {(y)} ,\sum\nolimits_{n = 1}^{n} {(z)} } \right)}}{n}$$

3 $${\text{If}}:a_{i} = \sum {x_{i} - \bar{x}} ,\,b_{i} = \sum {y_{i} - \bar{y}} ,\,c_{i} = \sum {z_{i} - \bar{z}}$$

4 $${\text{Then}}:A \cdot A^{T} = \left[ {\begin{array}{*{20}c} {\sum {a_{i}^{2} } } & {\sum {a_{i} b_{i} } } & {\sum {a_{i} c_{i} } } \\ {\sum {b_{i} a_{i} } } & {\sum {b_{i}^{2} } } & {\sum {b_{i} c_{i} } } \\ {\sum {c_{i} a_{i} } } & {\sum {c_{i} b_{i} } } & {\sum {c_{i}^{2} } } \\ \end{array} } \right]$$

To test the inter-observer reliability of the prototype surgical tracking system which was used to measure the prosthetic component orientations used in the range of motion simulation, two surgeons measured the landmarks of the APP for a total of 22 patients. The difference between the acetabular cup operative inclination and anteversion defined by Murray [23] was then compared relative to each surgeon’s measurement of the pelvic APP. The Pearson correlation coefficient for acetabular operative inclination was *r* = 0.93 (95 % CI 0.841–0.971) and for operative anteversion, *r* = 0.96 (95 % CI 0.905–0.983). To test the validity of using the AEP plane to define the neutral rotation of the femur to provide a reliable reference from which to construct the anatomical neutral posture for the subsequent range of motion simulation, 18 male subjects having a mean age of 31.5 years (24–42 years) were recruited for a motion analysis experiment using a Vicon MX motion capture system (*Vicon, Oxford, UK*). For each subject, the pelvic and femoral (using the AEP plane) coordinate frames were constructed and the alignment between the pelvic and femoral medial–lateral axes was measured in the transverse plane. A mean deviation of 0.38° (*σ* = 1.06°) was measured between the two axes which meant the AEP plane could be used to define the neutral rotation of the femur. Consequently, the constructed femoral coordinate frame could reliably be aligned with the coordinate frame of the pelvis to create a valid anatomical neutral posture [16, 29].

### Statistical analysis

A single replicate factorial design was used to assess which implant parameters, and their combined effects had the most significant influence upon both the size and position of the prosthetic motion area. A systematic fractional replicate design was first used to screen out those independent variables which were not main factors in influencing the impingement free range of motion [5]. This 2*k* + 2 design for *k* independent variables provided an estimate of the main effect $2{\text{Co}}_{j}$ and interaction ${\text{Ce}}_{j}$ for each independent variable. Consequently, the order of importance was estimated for the combined contribution by $\left( {M_{j} = \left\| {{\text{Co}}_{j} } \right\| + \left\| {{\text{Ce}}_{j} } \right\|} \right)$ [5]. The lowest factors were screened from the full factorial design. Two screening experiments were required for the modular neck group because the high (+) and low (−) values for neck ante–retroversion and varus–valgus were not able to be combined.

Following screening, a full factorial design of experiments was conducted with $2^{k}$experimental runs [22]. The estimated effect $\left( {E_{j} = \frac{n}{2}\sum\nolimits_{i = 1}^{n} {F_{ij} \cdot y_{i} } } \right)$ for each independent variable and their combined effects were calculated and ranked [22]. For the size of the prosthetic motion area in comparison with the healthy benchmark, those factors which had a greater than 1 % effect over the size of the prosthetic motion area were recorded. Considering the position of the prosthetic motion area relative to the healthy benchmark, those factors which had a greater than 1º effect over the three-dimensional angle were recorded.

Three experiments were performed for the modular neck group due to the fact that the maximum amount of 15º anteversion or retroversion could not be combined with the maximum 15º of varus or valgus. Consequently, the first experiment evaluated the combined effect between femoral neck ante–retroversion with femoral neck varus–valgus using the AR-VV necks which combine 4.5° of ante–retroversion with 6° of varus–valgus. The second and third experiments separately tested the effect of the maximum 15° ante–retroversion and varus–valgus necks with the other factors. This experimental design allowed for the contribution and interaction of femoral neck modularity, if identified as a main factor, to be fully evaluated against the other implant variables.

## Results

The systematic fractional replicate design to screen out independent variables which were not main factors in influencing the prosthetic impingement free range of motion found that in the non-modular group, femoral head offset (*M* 2.9 %, 1.0°), femoral neck offset (*M* 2.9 %, 1.0°) and femoral stem length (*M* 1.2 %, 1.0°) had the lowest contribution upon both the size of the prosthetic motion area and its position. For the modular neck group, in both screening tests, femoral head offset (*M* 5.5–6.3 %, 0.9–1.8°) and femoral neck length (*M* 2.0–2.5 %, 0.5–0.6°) had the smallest effect upon the prosthetic motion area. Consequently, these variables were screened from the full factorial experimental design.

**Fig. 3:**
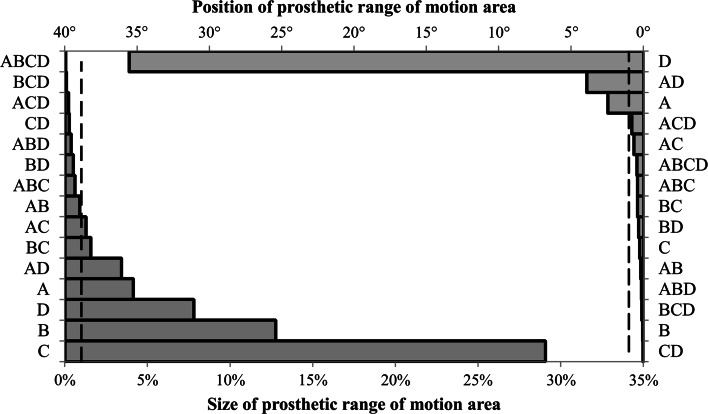
Ranking of estimated effect sizes for non-modular implant parameters on both the size (*percentage*) and position (*degree*) of the prosthetic motion area—(*A*) neck-shaft angle (*B*) femoral head diameter, (*C*) acetabular liner rotation centre depth, (*D*) prosthetic component orientation

The full factorial test for the non-modular control group using the main factors of femoral neck-shaft angle, femoral head diameter, acetabular liner rotation centre depth and prosthetic component orientation produced a 2^4^ factorial design. Figure 3 provides a Pareto chart of the estimated effect sizes for both the size of the prosthetic motion area and its position. Altering the acetabular liner rotation centre depth (*E* = 29.1 %) and femoral head diameter (*E* = 12.8 %) produced the largest change in the size of the prosthetic motion area, while prosthetic component orientation had the largest effect upon its position (*E* = 35.5°).

The first full factorial experiment in the modular neck intervention group which used the AR-VV modular necks produced a 2^5^ factorial design with the following variables—femoral neck varus–valgus, femoral neck ante–retroversion, femoral head diameter, acetabular liner rotation centre depth and prosthetic component orientation. There were in total eight factors found to have a greater than 1 % influence over the size of the prosthetic motion area with femoral head diameter (*E* = 18.4 %) and acetabular liner rotation centre depth (*E* = 16.7 %) having the largest effect upon the prosthetic motion area, while prosthetic component orientation had the largest influence upon its position (*E* = 25.6°). The interactive effect between prosthetic component orientation and femoral neck ante–retroversion had a modest effect upon the position of the prosthetic motion area (*E* = 6.2°).

**Fig. 4:**
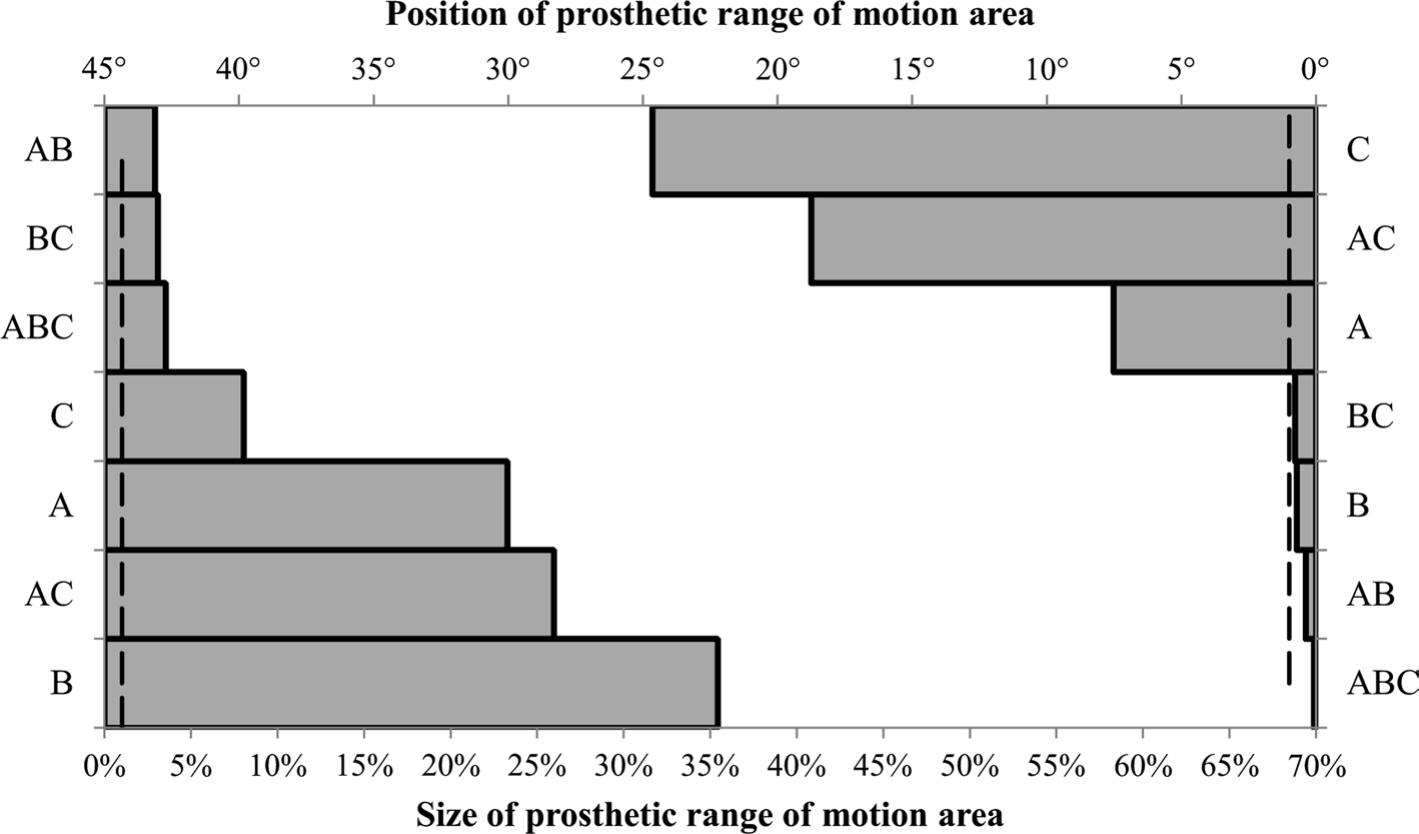
Ranking of estimated effect size for 15° ante-retroverted modular neck implant parameters on both the size (%) and position (°) of the prosthetic motion area—(*A*) femoral neck ante–retroversion, (*B*) femoral head diameter/acetabular liner rotation centre depth, (*C*) prosthetic component orientation

The second full factorial test analysed the effect of maximum 15° neck ante–retroversion. For this test, the factors of acetabular liner rotation centre depth and femoral head diameter were merged into a single factor. They were merged as the first full factorial experiment found they had no interactive effect and only influenced the size of the prosthetic range of motion area. Figure 4 shows that the 2^3^ factorial design resulted in all main and combined effects having a greater than 1 % effect upon the size of the prosthetic motion area with the diameter of the femoral head and the acetabular liner rotation centre having the greatest effect (*E* = 35.4 %). There were three factors which affected the position of the prosthetic motion area by greater than 1°—prosthetic component orientation (*E* = 24.6°), prosthetic component orientation × femoral neck ante–retroversion (*E* = 18.7°) and femoral neck ante–retroversion (*E* = 7.5°).

**Fig. 5:**
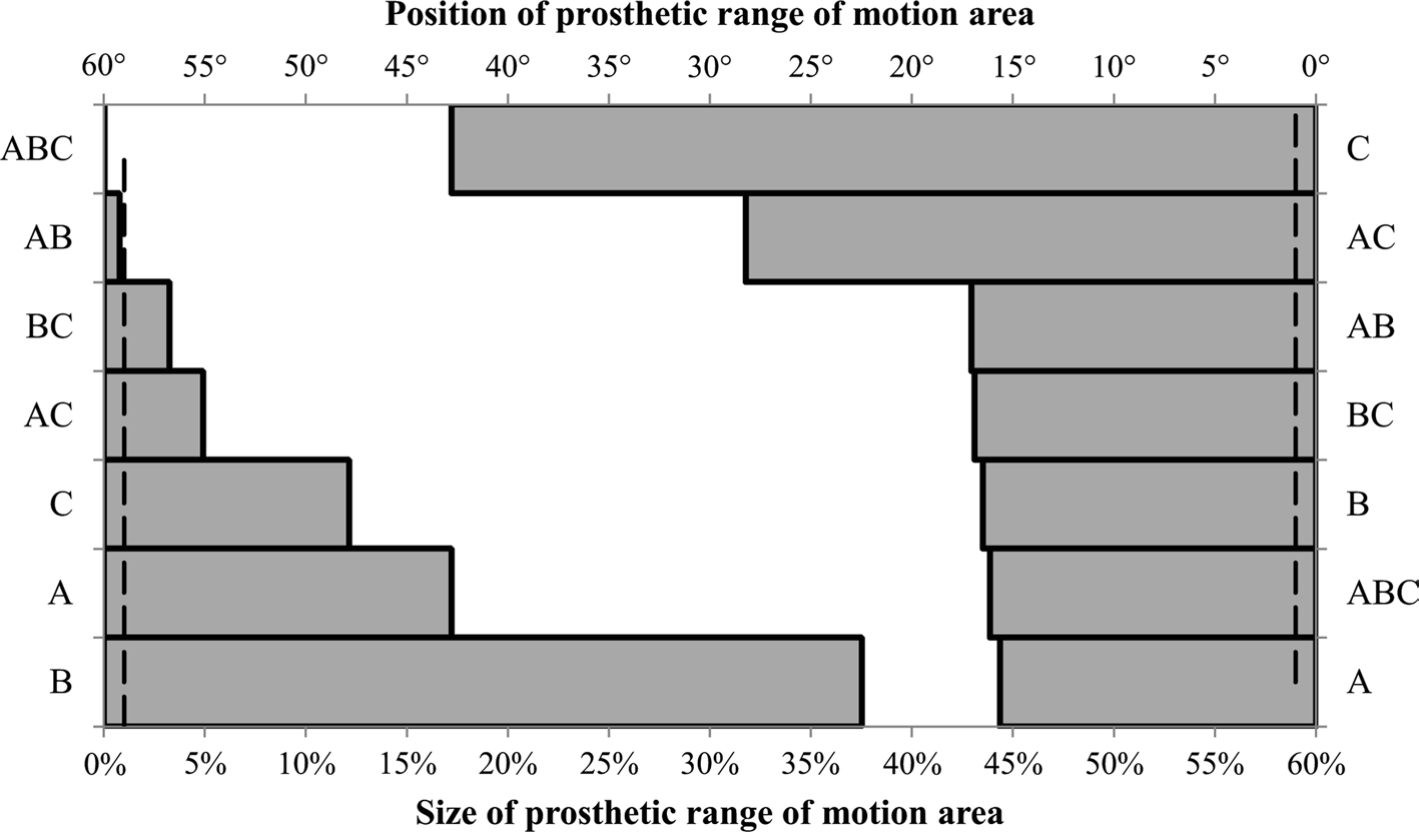
Ranking of estimated effect size for 15° varus–valgus modular neck implant parameters on both the size (%) and position (°) of the prosthetic motion area—(*A*) femoral neck varus–valgus (*B*) femoral head diameter/acetabular liner rotation centre depth, (*C*) prosthetic component orientation

Figure 5 shows the results for the final full factorial test which analysed the effect of maximum 15° neck varus–valgus. Six out of the seven factors had an estimated effect of greater than 1 % of the prosthetic motion area. The diameter of the femoral head in conjunction with the acetabular liner rotation centre depth (*E* = 37.5 %) as well as femoral neck varus–valgus (*E* = 17.2 %) had the largest effect. For the position of the prosthetic motion area, all seven factors had a greater than 1° influence, with prosthetic component orientation having the largest effect (*E* = 42.8°) followed by the combined effect of prosthetic component orientation × femoral neck varus–valgus (*E* = 28.2°).

## Discussion

In cementless THA, the surgeon has limited flexibility to control the degree of anteversion of the femoral stem [17]. Femoral neck modularity has been posed as a solution for this problem by providing independent adjustment of the version angle of the prosthetic femoral neck, as well as neck varus–valgus. However, there have been concerns with regard to their integrity which relate to the taper fitting of the femoral neck onto the femoral stem, potentially causing excessive fretting and crevice corrosion to the modular neck [1, 12]. Therefore, there needs to be an evaluation of whether there are any potential benefits to using a modular femoral neck in comparison with existing THA solutions.

**Fig. 6:**
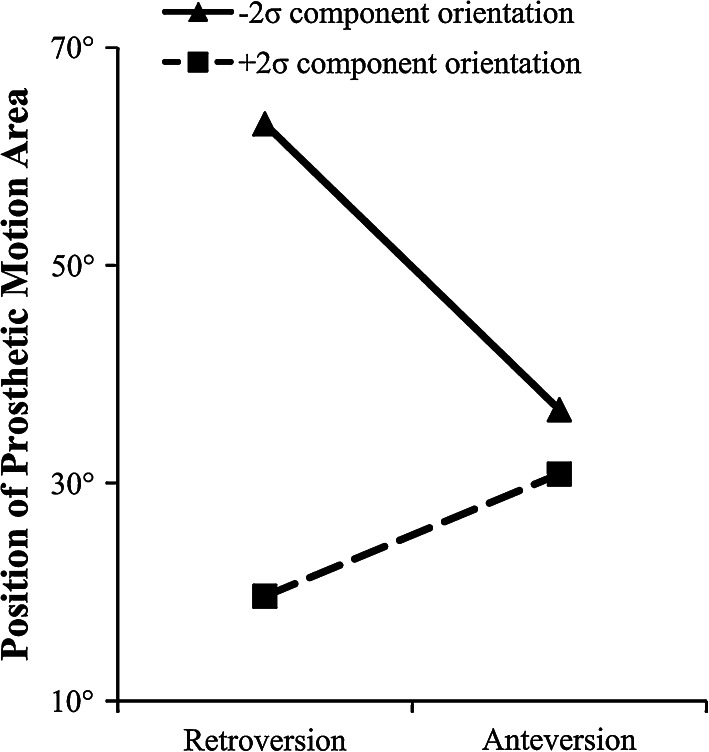
Interaction of the 15° ante–retroversion modular neck with component orientation upon the position of the prosthetic motion area

This study has found that the Profemur modular neck system, if altered to a large enough degree, can significantly increase the amount of prosthetic motion as well as alter its position to where it is required physiologically. Further, the way in which the modular neck system influences the prosthetic range of motion is different compared to the other modular parameters. Factors such as increasing femoral head diameter or reducing the depth of the acetabular liner rotation centre serve to increase the size of the prosthetic motion area by increasing the implant oscillation angle [55]. In contrast, femoral neck modularity, particularly with regard to femoral neck anteversion, is the only modular parameter which can alter the position of the prosthetic range of motion area. This can offset the effect of poor prosthetic component orientation by bringing the prosthetic range of motion back into alignment to where it is required physiologically. This is illustrated using the interaction diagram shown in Fig. 6, where femoral neck anteversion can be used to improve the alignment of the prosthetic motion area with the physiological requirement in cases where there is a low degree of cup anteversion, while the reverse is true for retroverted necks. Therefore, given a certain prosthetic orientation, femoral neck modularity is able to significantly improve a patient’s impingement free range of motion. However, in cases of severe acetabular cup mal-orientation, the study findings support those of Sakai et al. [30, 31] who found that the degree of correction offered by the 15° modular neck is not enough to bring the prosthetic motion area into alignment with the physiological requirement. Hence, there is a practicable limit that a modular femoral neck can correct [20, 30, 31].

**Fig. 7:**
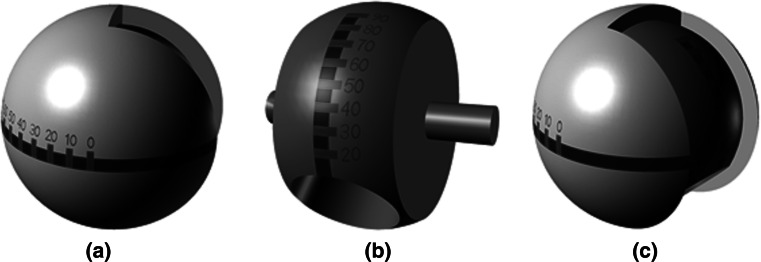
Trial femoral head design **a** combined anteversion measurement, **b** combined inclination measurement *and* **c** trial femoral head assembly

In traditional THA implants, other than increasing the femoral head-neck ratio there is very little opportunity to influence the range of motion to impingement. Recently, it has been shown that increasing the femoral head diameter to maximise the oscillation angle increases the risk of femoral neck fracture in hip resurfacing and has been associated with failures in metal-on-metal implants [25, 33]. Further, a larger femoral head may not be practicable for many patients, which means that achieving the correct prosthetic component orientation is vital to operative success. The results of this study have shown that femoral neck modularity is able to significantly improve a patient’s range of motion until impingement. However, given their effect, if the wrong neck choice is made, then this can have negative consequences with regard to range of motion until impingement. Making the correct neck choice may present difficulties to the surgeon with regard to assessing the amount of correction required. Therefore, there is a requirement to provide surgeons with better information to assess component orientation to be able to select the best femoral neck option. There are a number of options for this, either through the use of surgical navigation, CT pre-surgical planning or through the use of patient specific 3D printed templates. One such medical device is a trial femoral head shown in Fig. 7 with markings on its surface which can be used to inform about the combined version of the femoral neck as well as the additive angle of the femoral neck axis away from the transverse plane and acetabular cup inclination, termed combined inclination [47]. When the femoral head is located in the acetabular cup and the leg is placed in the anatomical neutral position, the surgeon is able to read these two angles from where they intersect with the rim of the acetabular cup. From this reading, the surgeon is then able to assess the amount of correction required and make the correct femoral neck selection [42].

There are a number of study limitations which are required to be noted. Firstly, only prosthetic impingement was considered in this study. There are other impingement modalities such as bone-on-bone and prosthetic-on-bone impingement [14, 21, 35]. It has been found that offset is more important with regard to bone-on-bone impingement than has been found in this study. Hence, it should be considered as an important factor in restoring a healthy range of motion during THA [14]. Secondly, this study has evaluated the Profemur modular neck and the Corail non-modular cementless stems, as well as their respective implant options. Consequently, the detailed study findings are specific to these two implants due to their individual implant designs. However, many of the modular implant options, such as choice of femoral head diameter, are common across implant manufacturers. Therefore, the findings of this study can be used to assess the effectiveness of femoral neck modularity in comparison with other modular parameters. However, the experimental design does not have the resolution to be able to draw conclusions about the subtleties of prosthetic design, such as the chamfer design of the acetabular cup or the exact geometry of the femoral neck. However, these were limited factors with regard to range of motion until impingement in comparison with the size of the femoral head or the degree of femoral neck version.

As well as the clinical and engineering investigations regarding the effectiveness of modular neck femoral stems. There have been concerns with regard to their integrity which relate to the taper fitting of the femoral neck onto the femoral stem, potentially causing excessive fretting and crevice corrosion to the modular neck [1, 12]. Corrosion of the stem–neck interface has been documented as causing inflammatory tissue reactions or catastrophic fracture of the femoral neck, with all documented cases requiring revision [46, 50, 51]. Consequently, there are other acknowledged risks regarding femoral neck modularity which must be considered within the context of the potential benefits highlighted by this study.

This study has found that femoral neck modularity is one of the main factors which can affect the amount of prosthetic motion a patient can achieve post-operatively. Indeed without this feature, once the acetabular cup and femoral stem orientations are fixed there is no further option to alter the prosthetic range of motion to a position where it is required physiologically. However, there are acknowledged risks to using these types of devices and the significance of making the incorrect neck selection could potentially be severe.

## Electronic supplementary material

Below is the link to the electronic supplementary material.

Supplementary material 1 (MPEG 27510 kb)

## Abbreviations

AEP: Ankle Epicondyle Piriformis Plane
APP: Anterior Pelvic Plane
CAD: Computer-aided design
CT: Computer tomography
*E*: Estimated effect size
*M*: Estimated combined effect size
THA: Total hip arthroplasty
